# Consensus between healthcare professionals on the “appropriateness” of attendances in an Irish emergency department

**DOI:** 10.1007/s11845-025-03961-0

**Published:** 2025-05-02

**Authors:** Conor Prendergast, John Ryan, Louise A. Barry, Rose Galvin, Niamh M. Cummins

**Affiliations:** 1 Emergency Department, St Vincents University Hospital, Dublin, Ireland; 2 Department of Nursing and Midwifery, Faculty of Education and Health Sciences, University of Limerick, Limerick, Ireland; 3 Ageing Research Centre, Health Research Institute, University of Limerick, Limerick, Ireland; 4 School of Allied Health, Faculty of Education and Health Sciences, University of Limerick, Limerick, Ireland; 5 School of Medicine, Faculty of Education and Health Sciences, SLÁINTE Research and Education Alliance in General Practice, Primary Healthcare and Public Health, University of Limerick, Limerick, Ireland; 6 Department of Paramedicine, Faculty of Medicine, Nursing and Health Sciences, Monash University, Melbourne, VIC Australia

**Keywords:** Alternative care pathways, Crowding, Emergency department, Healthcare professionals

## Abstract

**Background:**

Non-urgent care attendances to the emergency department (ED) increase the strain on this sector. Identification of patients who may benefit from appropriate alternative care pathways may serve to lessen ED crowding. However, healthcare professionals from different specialties may differ in their opinion of what is an appropriate use of ED services.

**Aim:**

The study aims to establish the consensus between healthcare professionals, from different specialties, on the appropriateness of attendances to an Irish ED.

**Methods:**

This was a single centre, cross-sectional study. Data were compiled in anonymised patient summary files (*n* = 77) from adults attending the ED over 24 h period. These summary files were reviewed by five different healthcare professionals; an emergency medicine consultant (EMC), an emergency medicine specialist registrar (EM SpR), an ED clinical nurse manager (CMN), an advanced paramedic (AP) and a general practitioner (GP). The clinicians were asked if the patient could have been managed by GP the same day or next day, if the patient’s ED visit was an inappropriate use of ED resources and to rank on a scale of 0–10 how appropriate the ED visit was.

**Results:**

Inter-rater agreement on management by GP in 24–48 h was 56% and ranged from 30% (CMN) to 40% (EMC). For inappropriate use of ED resources, consensus was 58% and ranged from 12% (GP) to 35% (EMC). Median “appropriateness” rating ranged from 6 (EM SpR) to 8 (AP and CMN). When the “appropriateness” scale was trichotomized, the “inappropriate” attendances ranged from 1% (CMN) to 21% (EM SpR), whilst “appropriate” attendances ranged from 47% (EM SpR) to CMN (78%).

**Conclusion:**

Despite agreement that there was “inappropriate” use of ED services, healthcare professionals from different backgrounds did not reach a general consensus on which attendances were inappropriate. Therefore, expectations regarding patients’ ability to self-assess illness or injury severity related to ED attendances may not be reasonable.

## Background

Emergency department (ED) attendances have continued to steadily rise in recent years, which have been accompanied by sharp increases in short-stay admissions and associated costs [1]. Increased ED attendances result in higher levels of crowding, which have been shown to directly contribute to poorer patient outcomes and staff inability to adhere to recommended treatment guidelines and standards [2]. Decreasing “low acuity” attendances to EDs may help to ease the growing issues of crowding and the burden of workload in EDs.

A previous Irish study found that potentially avoidable emergency presentations were primarily driven by socioeconomic conditions, hospital admission policies and health insurance coverage [3]. Internationally, it has been shown that patients attend their local ED for “low acuity” complaints for numerous reasons. These include trust in the hospital, perceiving their issue as very urgent, proximity of the hospital, expediency of being seen by a doctor and referral from another doctor to the ED [4]. A UK study that looked at over 3000 patients across 12 EDs reported that 15% of patients attending the ED were suitable for delayed treatment with their GP within 24 h [5, 6]. By targeting these “inappropriate” attendances to the ED, potential pathways could be put in place to decrease the burden on EDs. An inappropriate attendance at the ED by a patient can be defined as when their medical condition does not warrant an ED visit [7].

In Ireland, the all-of-government Slaintecare Action plan aims to improve population heath by delivering “the right care, in the right place, at the right time, by the right team” [8]. Central to this plan is a shift of the bulk of health services from an acute setting to the community. A reduction of inappropriate or avoidable attendances in the ED is a priority area for intervention by policymakers designing alternative care pathways [9]. However, consensus on appropriateness of ED attendances and the circumstances in which alternative care is practical has proved challenging for the academic and clinical community [10]. Not all healthcare professionals agree on what is deemed an “inappropriate” attendance to the ED and what qualifies as an unnecessary use of resources. The aim of this study was to look at the consensus between different healthcare specialties who work in and refer to urgent and emergency care services on the “appropriateness” of ED attendances over a 24-h period at one urban ED in Ireland. Clinicians from the disciplines of emergency medicine, emergency nursing, paramedicine and general practice were asked to rate the “appropriateness” of ED attendances.

These professionals were chosen as they represent those on the forefront of the emergency services here in Ireland but also as they represent staff from both an in-hospital and a pre-hospital setting. This study aims to clarify if professionals working in the community such as a paramedic or general practitioner deem certain patient attendances more or less “appropriate” when compared to their colleagues working in hospitals. However, many of these patients may not require urgent care but they may be deemed an appropriate referral due to the lack of alternative care pathways in the community [11].

## Methods

### Aim

The aim of this study was to establish the consensus between healthcare professionals, from different specialties, on the appropriateness of attendances to an Irish ED.

### Design

This was a cross-sectional study on a single centre in an urban ED in Ireland utilising data from the Better Data, Better Planning (BDBP) Study [12].

### Setting

Following ethical approval, data was collected from the ED of St. Vincent’s University Hospital (SVUH) over a single 24 h period in September 2020. The ED of SVUH serves a population of over 300,000, treating over 55,000 emergency admissions annually [13].

### Participants and procedure

**Fig. 1:**
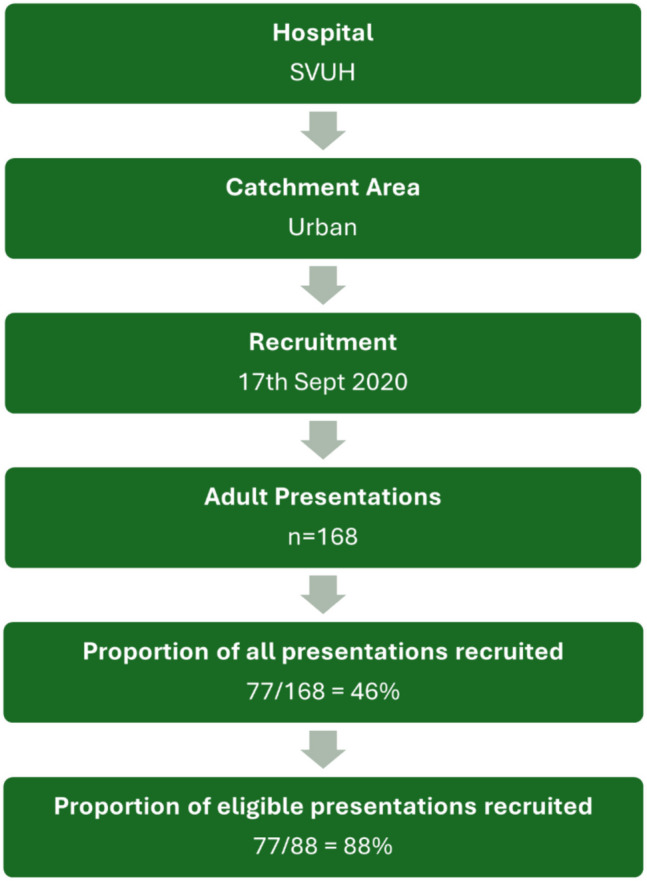
Flow chart of the study population (*n* = 77)

All adults attending the ED over the 24 h census period were eligible for inclusion. The inclusion criteria were as follows: (i) adults aged 18 and over (ii) a Manchester triage category [14] of 2–5 and the patient being medically stable with regards to heart rate, blood pressure, oxygenation, respiratory rate, temperature and mental status, (iii) patient had capacity and agreed to participate in the study. The following were the exclusion criteria: (i) scheduled ED admissions, (ii) patients with an altered mental status secondary to drug or alcohol intoxication, (iii) mental health presentations, (iv) inability to communicate in English. Of the *n* = 168 patients that attended the ED on Sept 17^th^ 2020, a total of *n* = 77 (46%) were deemed eligible for data collection (Fig. 1).

### Chart review

Data was compiled in patient summary files (*n* = 77) and were reviewed by five different healthcare professionals: an emergency medicine consultant (EMC), an emergency medicine specialist registrar (EM SpR), an emergency department clinical nurse manager (CMN), an advanced paramedic (AP) and a general practitioner (GP).

### Measure of “appropriateness” and rating scale analysis

The healthcare professionals were asked: (1) could the patient have been managed by a GP within the next 24–48 h; (2) was this attendance an inappropriate use of ED resources; (3) to rate the appropriateness of the attendance on a scale from 0 to 10. For the latter scale, a score of 0–3 was deemed to be an inappropriate attendance, 4–6 was deemed to be neither inappropriate or appropriate, whilst a score of 7–10 was considered an appropriate attendance.

### Data analysis

Data were entered into Excel, coded for analysis and analysed on SPSS (IBM SPSS Statistics Version 26). Variables were tested for normality using the Kolmogrov-Smirnov test. Continuous variables are presented as mean (standard deviation; SD) or median (interquartile range; IQR), according to distribution. The proportion of attendances in each category was calculated and cross tabulated for percentage agreement (consensus). Categorical data are presented as frequencies and percentages and chi-square test was applied to examine relationships between variables. Inter-rater agreement was calculated using Fleiss’ Kappa and presented with confidence intervals. A *p*-value of <0.05 was considered statistically significant.

## Results

### Demographics

The median age of the study population was 55 years (IQR 37–72 years) and the age range was 18–92 years (Table 1). In relation to gender, females comprised 53% and males comprised 47% of participants. Most participants attending the ED had health insurance (61%) and the majority travelled to the ED by private car (66%). The most frequent presenting complaint was musculoskeletal at 22%, followed by cardiovascular at 20% and trauma at 9% (Table 2). Most participants (39%) reported a duration of presenting complaint of <1 day but similar numbers (36%) reported having symptoms for >7 days prior to presentation. Most presentations were triaged as urgent (44%), the median length of stay in the ED was 4.5 h (IQR 2.5–6.9) and most patients were discharged following treatment (70%).

**Table 1:** Demographic and clinical characteristics of participating ED patients in SVUH (*n* = 77)

Category	Variable	Patients *n* = 77%
Gender	Female	53%
	Male	47%
Age Age category	Median, IQR	55, 37–72
	Range	18–92
	18–39 years	29%
	40–64 years	36%
	65 years+	35%
Healthcare coverage	Public—no cover	3%
	Public—medical card	36%
	Private insurance	61%
Mode of transport to ED	Ambulance	25%
	Private car	66%
	Public transport	7%
	Walk	3%
Presenting complaint*	Musculoskeletal	22%
	Cardiovascular	20%
	Gastroenterological	17%
	Trauma	9%
	Respiratory	7%
	Neurology	5%
	Nephrology	5%
	Ear, nose and throat	4%
	Other presentations	11%
Duration of complaint	<1 day	39%
	1–2 days	8%
	3–7 days	17%
	>7 days	36%
Triage category	Very urgent	30%
	Urgent	44%
	Standard	25%
	Non-urgent	1%
Length of stay	Median (h)	4.5
	IQR	2.5–6.9
	Range	0.0–14.4
	<1 h	12%
	1–2 h	7%
	2–4 h	23%
	4–8 h	43%
	8–16 h	16%
Disposition	Admitted	29%
	Discharged	70%
	Did not wait	1%

^*^Presenting complaint was characterised as per the categories outlined in the RCEM syllabus in the UK, which is aligned with the Irish Association of Emergency Medicine (IAEM) Training Standards in Ireland

### Consensus measures

Consensus on whether the patient could have been managed by their GP in 24–48 h ranged from 30% (CMN) to 40% (EMC) with an overall consensus of 56%. The inter-rater agreement was *κ* = 0.511 (*p* < 0.001), demonstrating a moderate agreement (Table 2).

**Table 2:** Inter-professional consensus on appropriate attendances to the ED (*n* = 77)

Variable	Advanced paramedic (*n*, %)	Clinical nurse manager (*n*, %)	General practitioner (*n*, %)	Specialist registrar (*n*, %)	Emergency consultant (*n*, %)	Inter-rater agreement (*n*, %) Kappa CI *p*-value
Management by GP in 24–48 h	24, 31%	23, 30%	27, 33%	30, 39%	31, 40%	43, 56%
						0.511
						(0.440–0.582) *p* < 0.001
Inappropriate use of ED resources	17, 22%	10, 13%	9, 12%	24, 31%	27, 35%	45, 58%
						0.435
						(0.363–0.506) *p* < 0.001

In terms of inappropriate use of ED resources, consensus ranged from 12% (GP) to 35% (EMC) with an overall consensus of 58%. The inter-rater agreement was *κ* = 0.435 (*p* < 0.001), demonstrating a moderate agreement (Table 2).

The median score for rating “appropriateness” of ED attendance ranged from 6 (EM SpR) to 8 (AP and CMN). The inter-rater agreement was *κ* = 0.113 (*p* < 0.001), demonstrating poor agreement (Table 3). When the “appropriateness” rating scale was trichotomized, the overall consensus across professions was 40% (1% inappropriate, 3% neutral, 36% appropriate). The inappropriate attendances ranged from 1% (CMN) to 21% (EM SpR), whilst appropriate attendances ranged from 47% (EM SpR) to CMN (78%). Those deemed “neither inappropriate or appropriate” ranged from 21% (CMN) to 33% (EM SpR) (Table 3).

**Table 3:** Inter-professional consensus on appropriate attendances to the ED according to a rating scale of appropriateness (*n* = 77)

Variable	Advanced paramedic (*n*, %)	Clinical nurse manager (*n*, %)	General practitioner (*n*, %)	Specialist registrar (*n*, %)	Emergency consultant (*n*, %)	Inter-rater agreemen**t** (*n*, %) Kappa CI *p*-value
Appropriateness rating scale 1–10	Median 8 IQR 5–10	Median 8 IQR 7–10	Median 7 IQR 5–8	Median 6 IQR 4–9	Median 7 IQR 5–7	0.113 (0.085–0.141) *p* < 0.001
Inappropriate attendance^a^ (appropriateness rating scale, 0–3)	7, 9%	1, 1%	8, 11%	16, 21%	13, 17%	1, 1%
Neither appropriate nor inappropriate attendance^a^ (appropriateness rating scale, 4–6)	21, 27%	16, 21%	22, 29%	25, 33%	17, 22%	2, 3%
Appropriate attendance^a^ (appropriateness rating scale, 7–10)	49, 64%	60, 78%	46, 61%	36, 47%	47, 61%	28, 36%

^a^Derived from trichotomisation of the rating scale

## Discussion

The results of this study demonstrate that healthcare professionals from different specialities agree that there is “inappropriate” use of EDs in Ireland; however, there was not a clear consensus on which particular attendances were “inappropriate” or avoidable.

With regards to whether the patient could be managed by the GP within the next 24–48 h, there was a 56% consensus. The range variance was low with the highest being 40% (EMC) and lowest being 30% (CMN). The healthcare professionals who aligned most closely and who felt that more patients could have been dealt with at a primary care level was the emergency medicine consultant (40%) and SpR (39%). It would be expected that that doctors working in the same specialty should have a similar outlook on this having undergone the same core and advanced training schemes in emergency medicine [15]. Furthermore, considering their speciality, emergency doctors are likely to perceive less acute attendances as non- emergencies as they are experienced in dealing with high acuity emergencies on a regular basis. GPs who tend to see lower acuity patients in primary care deemed a lower percentage (33%) could have been managed at a primary care level. This is similar to the findings of a recent Australian study which estimated that up to 27% of ED patients were potentially suitable for GP care [16].

The lack of consensus across professions was more obvious when it came to the inappropriate use of ED resources. The range here was wider with the GP deeming 12% of attendances inappropriate whilst the EMC deemed 35% inappropriate. This supports previous findings from the BDBP study [17] and may reflect different opinions but also a lack of understanding from both parties of the constraints on both services. For example the GP may not have access to alternative care pathways so deem a patient appropriate for referral to the ED, whilst an EMC may be aware that for particular patients an ED attendance does not provide any benefit or expedite waiting times for treatments or investigations. This is an area that may be of benefit to further delve into for future studies.

Our findings are in agreement with the findings of a previous Irish survey investigating healthcare providers’ perceptions of the appropriateness of ED attenders [18]. A similar New Zealand study also reported significant differences in the attitudes and perceptions of healthcare professionals involved in the referral, treatment, and admission of patients to the ED [19]. These studies also demonstrated the difficulty of establishing a clear consensus between healthcare staff on the type of attendances that are deemed appropriate.

When participants were asked to rate the appropriateness on a scale there was a poor inter-rater agreement. Trichotomisation of the rating scale into categories related to “appropriateness” of attendance demonstrated large differences in opinion across specialities. For example, the CMN only classified one of the attendees to be an inappropriate attendance whilst the SpR rated 16 (21%) of attendances as inappropriate. Similarly with regard to appropriate attendances, the CMN rated 78% of attendances as appropriate whilst the EM SpR rated only 47% as appropriate. These professionals work together in the same area so the large discrepancy in opinion on appropriateness of attendance is surprising but only further adds to the conclusion that obtaining a general consensus from healthcare professionals on this topic is extremely difficult. Considering professionals, with insight into how the healthcare framework operates find it hard to concur on what is an inappropriate attendance is, patients without this knowledge, cannot be expected to attend the service most appropriate to them.

A recent meta-analysis comparing triage levels in the ED between nurses and senior physicians reported moderate agreement (*k* = 0.683; 0.546–0.748) on paper-based scenarios but higher concordance was observed between the professions when reviewing live cases *k* = 0.793 (95% CI, 0.679–0.869). The paper-based care review is one of the limitations of our study.

Another limitation is the fact that this was a cross-sectional study in a single centre over a 24 h period. Further studies would benefit if there was a larger sample size across many sites to improve external validity. The study also took place in 2020 during the COVID-19 pandemic which may have impacted on the findings. Additionally, the sample size was small with just one representative from each healthcare speciality performing the chart review, if power was increased and more delegates from each speciality participated it may help prevent potential bias. Finally, another source of potential bias arises from the perception that healthcare professionals may be more likely to deem an attendance inappropriate in theory but may approach the same case clinically with a different outlook.

There is further work to be done in this field of study. The lack of consensus between primary care and emergency care services may be rooted in poor communication of what pathways are available to each. More structured feedback from emergency care services to GPs on what the ED can and cannot provide may facilitate an improvement in this. This study also only focused on staff working in the prehospital setting or in the ED. However inappropriate attendances can also arise from inpatient team using the ED for resources such as faster access to investigations, amongst others. It would be interesting to delve further into this area.

## Conclusions

Overall, this study showed that despite agreement that there was “inappropriate” use of the ED services, healthcare professionals from different backgrounds only had a general consensus of just over 50% on which attendances were inappropriate. Healthcare professionals struggle to agree on what is an appropriate use of the emergency services, despite their perceived insight in the healthcare management framework. Therefore, the general public cannot be expected to have a greater understanding of what is an inappropriate use of the emergency services. In the absence of other interventions, our current high ED attendances are likely to continue to escalate.
